# Nanopore sequencing enables combined detection of *USP7* variants and a known Hao-Fountain syndrome episignature

**DOI:** 10.3389/fgene.2025.1730165

**Published:** 2026-01-05

**Authors:** Liselot van der Laan, Martin A. Haagmans, Andrea Venema, Jennifer Kerkhof, Michael A. Levy, Silvana Briuglia, Pilar Caro, Sebastian Sailer, Christian P. Schaaf, Bekim Sadikovic, Mieke M. van Haelst, Mariëlle van Gijn, Mariëlle Alders, Peter Henneman

**Affiliations:** 1 Department of Human Genetics, Amsterdam UMC, Amsterdam, Netherlands; 2 Amsterdam Reproduction & Development, Amsterdam University Medical Centers, University of Amsterdam, Amsterdam, Netherlands; 3 London Health Science Centre, Verspeeten Clinical Genome Centre, London, ON, Canada; 4 Department of Pathology and Laboratory Medicine, Western University, London, ON, Canada; 5 Department of Biomedical and Dental Sciences and Morphofunctional Imaging, University of Messina, Messina, Italy; 6 Institute of Human Genetics, Heidelberg University, Heidelberg, Germany

**Keywords:** DNA methylation, episignature, Hao-Fountain syndrome, nanopore, *USP7*

## Abstract

**Background:**

Hao-Fountain syndrome (HAFOUS) is a rare neurodevelopmental disorder caused by pathogenic variants in the *USP7* gene. This condition is associated with a distinct DNA methylation episignature that aids its diagnosis. While microarray-based methods have traditionally been used to detect these DNA methylation signatures, long-read nanopore sequencing offers the potential for simultaneous genetic and epigenetic analysis.

**Methods:**

We analyzed DNA extracted from the blood of five individuals carrying pathogenic *USP7* variants or deletions using Oxford Nanopore direct long-read sequencing. This approach enabled the detection of both genetic variants and native 5 mC methylation profiles. Methylation patterns were analyzed at known HAFOUS-specific episignature probes and compared against control samples using UMAP and hierarchical clustering. Classification was further validated using the EpiSign™ platform.

**Results:**

Nanopore sequencing successfully identified all pathogenic *USP7* variants, including SNVs and structural deletions. DNA methylation analysis demonstrated clear separation between HAFOUS patients and controls, consistent across both nanopore and EPIC array platforms. All cases were correctly classified using the EpiSign™ pipeline, confirming the presence of the HAFOUS episignature.

**Conclusion:**

This study demonstrates that nanopore sequencing enables accurate, simultaneous detection of *USP7* variants and the associated HAFOUS methylation episignature. These findings support the clinical utility of long-read sequencing as an integrated diagnostic tool for neurodevelopmental disorders, offering a unified platform for comprehensive genomic and epigenomic profiling.

## Introduction

Hao-Fountain syndrome (HAFOUS) is a rare neurodevelopmental disorder caused by pathogenic variants in the *USP7* gene (Ubiquitin-Specific-Processing Protease 7; OMIM #602519) or by chromosomal deletions encompassing *USP7*. Clinically, HAFOUS is characterized by developmental delay, intellectual disability, speech impairment, behavioral abnormalities, autism spectrum disorder, seizures, hypogonadism, and mild dysmorphic features (Fountain et al., 2019; Hao et al., 2015; Wimmer et al., 2024). *USP7*, located on chromosome 16p13.2, encodes a deubiquitinating enzyme involved in multiple cellular pathways, including chromatin remodeling and regulation of the MDM2–P53 pathway, which is critical for DNA repair, transcriptional control, and tumorigenesis (Li et al., 2002; Schaefer and Morgan, 2011; Nicholson and Suresh Kumar, 2011). Furthermore, *USP7* has recently been shown to be associated with a unique DNA methylation episignature, which provides a valuable biomarker for HAFOUS and can aid in the classification of uncertain genetic variants (van der Laan et al., 2023).

Episignatures are reproducible, genome-wide DNA methylation patterns that serve as disease-specific molecular fingerprints. Since 2019, they have increasingly been applied in diagnostic settings, particularly for disorders caused by defects in chromatin regulators and other components of the epigenetic machinery (Sadikovic et al., 2019; Levy et al., 2022). Currently, EpiSign can distinguish 99 neurodevelopmental and syndromic conditions, making it a powerful complementary diagnostic tool. These signatures are especially valuable in cases where genetic testing identifies variants of uncertain significance (Acharya et al., 2022) or where the clinical presentation is ambiguous. By comparing a patient’s methylation profile to established reference signatures, it is possible to confirm a diagnosis, refine genotype–phenotype correlations, and even identify novel syndromes. Importantly, EpiSign is generally used as a tier 2 test, following whole-exome or whole-genome sequencing. Consequently, patients often require two separate assays, which increases turnaround time and limits overall diagnostic efficiency. These limitations underscore the need for integrated approaches, such as nanopore sequencing, which can simultaneously assess both genetic variants and DNA methylation patterns in a single assay (Sadikovic et al., 2019; Kerkhof et al., 2024).

Nanopore sequencing offers a transformative opportunity to integrate genomic and epigenomic analyses in a single assay. Unlike traditional bisulfite-based methods to assess methylation status, direct nanopore sequencing detects both single nucleotide variants (SNVs) and native DNA methylation marks directly, without chemical conversion or amplification biases (Kolmogorov et al., 2023; Zhang et al., 2024). Recent proof-of-concept studies have demonstrated that long-read nanopore sequencing can identify disease-specific episignatures with accuracy comparable to microarray-based methods, while simultaneously resolving structural variants, imprinting defects, and X-chromosome inactivation patterns (Logsdon et al., 2020; Ebert et al., 2021; Giesselmann et al., 2019; Johansson et al., 2023; Yamada et al., 2023; Patel et al., 2022). A recent clinical study demonstrated that nanopore-based episignature detection in developmental disorders is both feasible and diagnostically valuable, effectively consolidating multiple genetic and epigenetic assays into a single comprehensive platform (Geysens et al., 2025). This integrated approach holds promise for other neurodevelopmental disorders (such as *USP7*), enabling the simultaneous detection of pathogenic variants and the associated methylation signature.

This study aims to define the DNA methylation episignature of Hao-Fountain syndrome using nanopore sequencing. We evaluate whether long-read sequencing can concurrently detect *USP7* variants and their associated methylation pattern, offering a unified approach for genetic and epigenetic characterization of HAFOUS.

## Materials and methods

### Study cohort

This study included blood samples from five individuals (one male and four females) carrying either pathogenic *USP7* variants or a chromosomal deletion encompassing the *USP7* gene (Table 1). Blood was chosen because the HAFOUS-specific episignature—and episignatures for all EpiSign episignature are robustly detectable in peripheral blood, providing a practical, minimally invasive, and clinically validated tissue source that reliably reflects disease-associated methylation changes (van der Laan et al., 2023; Aref-Eshghi et al., 2021). All patients underwent prior whole-exome sequencing at our ISO 15189 accredited clinical genome diagnostics laboratory (Amsterdam UMC, Human Genetics), confirming *USP7* as the sole pathogenic variants. All variants were classified based on the guidelines from the American College of Medical Genetics and Genomics (ACMG), the Association for Molecular Pathology (AMP), and the Human Genome Variation Society (HGVS) recommendations (Richards et al., 2015; Riggs et al., 2020; den Dunnen et al., 2016). All patients, except for Case 3, have been previously published in relation to the *USP7* episignature and clinical presentation (Wimmer et al., 2024; van der Laan et al., 2023; Capra et al., 2021). Case 3, although unpublished, also demonstrates a positive *USP7* episignature as determined using Illumina EPIC array-based DNA methylation profiling.

**TABLE 1:** Sequencing summary metrics for all samples.

Sample ID	Input (ng)	Load amount (ng)	Total yield (Gb)	Read N50 (kb)	Mean genome coverage (x)	≥20× coverage at USP7 (%)	Methylation calls with ≥ 20× coverage (#)
Case 1	960	500	111.9	7.722	35.56	98.4	25,766,739
Case 2	960	500	115.4	7.765	36.41	99.7	26,480,518
Case 3	960	500	130.5	6.154	41.47	80.2	27,081,383
Case 4	960	500	147.3	8.342	46.93	99.7	27,530,669
Case 5	960	500	124.1	7.132	39.52	99.8	27,001,157

### Sample preparation and nanopore sequencing

Genomic DNA was extracted from whole blood using standard protocols. DNA integrity was assessed using the Tape station 4200 (Agilent) and quantity was assessed using a Qubit 2.0 Fluorometer. DNA was fragmented to 10 kb using gTUBES (Covaris) following ONT’s ligation sequencing kit V14 protocol (GHD_9173_v114_revB_10Nov2022), starting with approximately 960 ng genomic DNA. For each sample a library was prepared using ONT’s Ligation Sequencing Kit V14 (SQK-LSK114). Libraries were quantified using a Qubit 2.0 Fluorometer, 500 ng was loaded on a R10.4.1 flow cell. Whole-genome sequencing was performed on a Promethion for 72 h, without additional reloading, generating long-read data for both variant detection and methylation analysis.

### Data analysis

5mCG super-accuracy (v5.0.0) base calling was performed using an in-house pipeline (v2.6.1 Human Genetics Amsterdam UMC/Genome Analysis/sushi · GitLab) that incorporates Dorado 0.9. Further analysis was conducted using another in-house pipeline (Human Genetics Amsterdam UMC/Genome Analysis/pika · GitLab), which also uses Dorado 0.9 for alignment. Structural variant calling was performed with Sniffles2 (v2.3.3) (Smolka et al., 2024), small variant calling with Clair3 (v1.0.8), and methylation calling with modkit (v0.4.3). To summarize, after basecalling reads were aligned to hg38 followed by small variant calling, structural variant calling and methylation calling.

### Clustering and episignature detection

DNA methylation calls overlapping Illumina Infinium MethylationEPIC BeadChip v2 array probes were extracted from Nanopore data using custom R scripts. For each sample, methylation beta values per position were calculated as the fraction of methylated reads divided by the total number of reads, and probe IDs were matched to a curated *USP7* episignature probe set. The curated *USP7* episignature probe set used in this study was adapted from the previously published HAFOUS methylation episignature by van der Laan et al. in Supplementary Table S2 (van der Laan et al., 2023) which was originally defined on the MethylationEPIC v1 array. In the current study, probe IDs were mapped to the corresponding probes on the v2 array, ensuring compatibility with the updated array an full reproducibility. Probe-level beta values from all *USP7* patient and control samples were merged using the dplyr R package (v1.8.9) to create a single matrix of methylation profiles. The resulting beta matrix was analyzed with uniform manifold approximation and projection (UMAP) using the umap R package (v0.2.10.0) (random_state = 15) to reduce dimensionality to two components. UMAP projections were visualized with plotly, with samples colored by group (*USP7* patient or control) and shaped by platform (Nanopore or MethylationEPIC array). Hierarchical clustering heatmaps of the same beta matrix were generated with the pheatmap package (v1.0.12) in R, including column annotations indicating sample group and platform. Clustering was performed using Euclidean distance and complete linkage. This analysis enabled visualization of the distinct *USP7* episignature and assessment of concordance between Nanopore-derived methylation profiles and controls.

### EpiSign™ confirmation using nanopore sequencing

To ensure compatibility with the Illumina array–based EpiSign™ v5 classification pipeline (London Health Sciences Centre, Canada), Nanopore-derived 5 mC calls were restricted to CpG sites represented on the Infinium MethylationEPIC v2 array. For each probe, β-values were calculated as the proportion of methylated reads (methylated/total). When methylation percentages were reported, these were converted to fractions (0–1). Probe-level β-values were then formatted to match array probe IDs and submitted for analysis using the clinically validated EpiSign v5 pipeline, following previously established methods (Levy et al., 2022; Kerkhof et al., 2024; Aref-Eshghi et al., 2021; Sadikovic et al., 2021). The support vector machine (SVM)-based model compares patient methylation profiles to the EpiSign Knowledge Database, which includes over 20,000 Illumina array reference samples. Nanopore sequencing cases were assessed using unsupervised hierarchical clustering and multidimensional scaling, and considered positive if they showed case–control separation similar to that seen in the array data.

## Results

### Sequencing

Sequencing yield was similar for all patients ranging from 107 Gb to 137 Gb with N50 ranging from 5.88 kb to 7.7 kb corresponding to a mean genome coverage of 35× to 47×. A detailed overview of per-sample sequencing performance is provided in Table 1.

### Genetic findings

Nanopore sequencing successfully identified and confirmed all previously known or suspected variants in the *USP7* gene across the five included cases (Table 2). The sequencing data confirmed the presence of four single nucleotide or small indel variants and one chromosomal microdeletion affecting the 16p13.2 region (Supplementary Figure S1).

**TABLE 2:** Patient table.

Case	Sex	Age	Variant NM_003470.3	ACMG-classificatie
1	M	9	c.2132_2140+9del	Likely pathogenic
2	F	20	c.1988A>C, p.(Glu663Ala)/c.2051A>T, p.(Asp684Val)	Likely pathogenic
3	F	8	arr[GRCh37] 16p13.2(9036896_9283604)x1	Pathogenic
4	F	11	c.1258A>G p.(Lys420Glu)	Likely pathogenic
5	F	10	c.2232_2235delGAGA p.(Arg745AsnfsTerS)	Likely pathogenic

### Platform correlation analysis

To quantitatively assess agreement between Nanopore and EPIC array platforms, we calculated Pearson correlation coefficients for β-values across the 179 HAFOUS episignature CpG sites available on both MethylationEPIC v1 (original signature: 203 probes) and v2 arrays. We observed that the correlation coefficients of control and case pairs, including both EPIC and Nanopore generated beta values, ranged between 0.75 and 0.88 (Supplementary Table S1). These strong correlations confirm the robustness of Nanopore-derived methylation profiles despite probe mapping differences between array versions.

### Quality control and primary data exploration

Initial exploration of DNA methylation beta values at *USP7* episignature probes was performed using UMAP to assess overall sample structure. A total of 179 CpG sites corresponding to the HAFOUS episignature were analyzed. All sites were successfully measured in each case and control sample, and the observed β-values for each probe across all samples are provided in Supplementary Table S1. This analysis indicated clear separation between *USP7* patient samples and controls, with all *USP7* samples clustering together and controls forming a distinct group, consistent across both Nanopore and EPIC array platforms. These results confirm that the episignature is detectable in both data types and support subsequent, more detailed analyses.

### Clustering and multidimensional scaling

**FIGURE 1:**
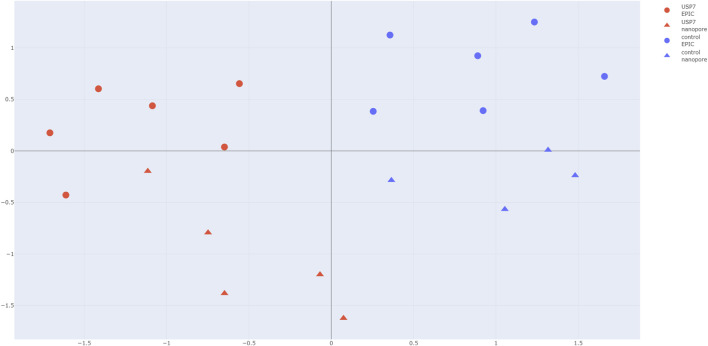
Comparative analysis of the *USP7* episignature in HAFOUS using Nanopore sequencing and Illumina EPIC array data. UMAP projection based on methylation β-values at HAFOUS/*USP7* episignature probes. *USP7* patient samples (red) and unaffected controls (blue) form distinct clusters. Circles represent EPIC array data; triangles represent Nanopore data. The clear separation by disease status, independent of platform, demonstrates the ability to detect the *USP7* episignature using Nanopore sequencing.

Hierarchical clustering and multidimensional scaling (MDS) of the same probe set further confirmed the separation between *USP7* patients and controls (Figure 1), providing a robust visualization of disease-specific methylation patterns across platforms.

### EpiSign™ confirmation using nanopore sequencing

**FIGURE 2:**
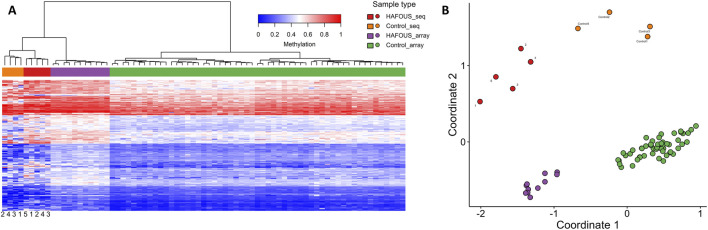
HAFOUS episignature analysis from peripheral blood: comparison of nanopore sequencing and Illumina EPIC array data in individuals with pathogenic *USP7* variants. **(A)** Hierarchical clustering and **(B)** multidimensional scaling plots show that HAFOUS nanopore sequencing cases (red) are distinct from Control nanopore sequencing cases (orange), with separation patterns consistent with those observed between HAFOUS EPIC- array cases (purple) and Control EPIC-array cases (green) (Vollger et al., 2025).

Episign™ variant-targeted analysis showed that HAFOUS and negative control cases, sequenced with Nanopore technology, separated in a pattern consistent with the array data, as demonstrated by Euclidean clustering and multidimensional scaling (Figure 2). The separation along coordinate 1 was the most pronounced distinction between patients and controls. Importantly, for both groups, Nanopore and EPIC array data clustered in the same regions, indicating strong concordance between platforms in capturing the HAFOUS episignature. This separation was restricted to the episignature probe set specific to HAFOUS, while probe sets for other syndromes (e.g., GADEVS) showed mixing between HAFOUS and Negative Control cases (Supplementary Figure S2).

## Discussion

In this study, we establish that long-read nanopore sequencing can accurately detect both pathogenic *USP7* variants and the DNA methylation episignature associated with HAFOUS. The nanopore based episignature showed herein similar separation and distances of cases and controls compared to EPIC-array, demonstrating equivalent robustness of the nanopore-based episignature. All cases demonstrated distinct methylation profiles separating them from controls in the clinically validated EpiSign™ pipeline. These results highlight the feasibility of using a single nanopore-based assay to capture both genetic and epigenetic hallmarks of HAFOUS.

While prior studies have defined the *USP7* episignature using microarray-based platforms, our findings confirm that long-read sequencing achieves comparable performance in identifying disease-specific methylation patterns. The separation along coordinate 1 was the most pronounced distinction between patients and controls, and Nanopore and EPIC array data clustered in the same regions for both groups, demonstrating strong concordance between platforms. This reinforces the clinical reliability of native methylation calling from Nanopore data and supports a shift toward streamlined, multi-modal molecular diagnostics that consolidate variant detection and methylation profiling into a unified workflow.

A key advantage of nanopore sequencing lies in its ability to directly sequence native DNA without bisulfite conversion, preserving epigenetic information across long contiguous reads (Logsdon et al., 2020). We envision that in a diagnostic setting for rare genetic disorders, methylation profiling can serve as an initial screening tool to indicate the presence of a syndrome before the causative gene is known. Subsequent whole-genome nanopore sequencing then enables simultaneous confirmation of pathogenic variants and comprehensive analysis of all variant types in a single assay, providing a complete molecular characterization in one experiment (Satam et al., 2023). Long reads also allow phasing of variants and the potential assessment of allele-specific methylation. Although allele-specific effects have not yet been reported in HAFOUS, this capability could be leveraged in future studies to explore complex genotype–epigenotype interactions. Such multi-modal data generation is particularly valuable for disorders like HAFOUS, where both genetic and epigenetic mechanisms contribute to disease etiology. Although long-read data enable allele-specific methylation and inter-patient comparisons, as previously shown by others [Akbari, 2021 #222][Kolmogorov, 2023 #9; Genner, 2025 #223], these were beyond the scope of this proof-of-concept study and may be explored in future work.

Several recent studies have explored the feasibility of nanopore-based episignature detection in other Mendelian disorders (Logsdon et al., 2020; Ebert et al., 2021; Giesselmann et al., 2019; Johansson et al., 2023; Yamada et al., 2023; Patel et al., 2022; Geysens et al., 2025). These proof-of-concept and clinical investigations have demonstrated that long-read sequencing can accurately identify disease-specific methylation patterns, in some cases rivaling or even surpassing microarray-based approaches. Our study adds to this growing body of evidence, specifically focusing on *USP7*-related neurodevelopmental disease. The consistent classification of all five cases, underscores the reliability of nanopore-derived episignature detection and supports its potential for broader clinical adoption, possibly as a Tier 1 diagnostic strategy integrating both variant detection and methylation profiling.

Despite these promising outcomes, some limitations warrant consideration. First, the cohort size was relatively small, reflecting the rarity of HAFOUS. Second, while nanopore-derived methylation calls aligned well with established reference signatures, platform-specific biases cannot be fully ruled out and should be evaluated in larger, more diverse datasets. Third, current episignature classification pipelines, including those used in this study, were originally developed for array-based data. As such, they may not be fully optimized for the read structure and depth variability of long-read sequencing. Further development of bioinformatics tools specifically tailored to long-read methylation data will be critical for maximizing diagnostic accuracy.

Additionally, biological factors such as mosaicism or skewed X-chromosome inactivation (XCI) may in principle influence detection of disease-specific methylation patterns. Although we did not specifically assess XCI or mosaicism in our cohort, future work could leverage the haplotype-aware and allele-specific methylation capability of nanopore sequencing to address such variables. Moreover, while tissue-specific methylation profiling may ultimately provide additional insights, this would require the availability of large-scale reference datasets across multiple tissues, which is currently not feasible for diagnostic practice (Geysens et al., 2025; Vollger et al., 2025; Oexle et al., 2023).

While episignature assays generally show good specificity, partial overlap between certain conditions or with not-yet-characterized signatures can occur, which may complicate classification (Levy et al., 2022). In addition, although this study focused on curated episignature probes to enable direct comparison with array-based data, native nanopore sequencing provides the unique opportunity to interrogate all CpG sites across genomic loci without restriction to predefined array probes. This capability allows for comprehensive methylation profiling over large contiguous genomic regions and single DNA molecules, preserving methylation context and enabling detection of allele-specific methylation and regulatory elements missed by microarray platforms. Recent comparative studies confirm ONT sequencing’s robust performance and enhanced resolution for genome-wide methylation analysis, highlighting its utility in detecting complex epigenetic patterns beyond episignature panels [de Abreu, 2025 #220]. Future work leveraging whole-locus native methylation data promises to improve the discriminative power of diagnostics, refined genotype-epigenotype correlations, and discovery of novel regulatory mechanisms. Microarray-derived reference data, often based on limited number of CpG sites, may reduce sensitivity when applied to sequencing-based methylomes. As such, the creation of large-scale, nanopore-based reference methylomes will be essential to refine existing episignatures, improve sensitivity and specificity, and facilitate the discovery of novel ones (Silva et al., 2022). Such references would also provide extensive whole-genome data that could be used to train new episignatures. In addition, in a future genome-diagnostic application based on well-defined episignature panels, Nanopore’s targeted enrichment strategy, i.e., adaptive sampling, could increase on-target read depth and potentially improve methylation-calling accuracy at loci of interest. Both approaches represent promising directions for future studies.

With continued advancements in basecalling accuracy, throughput, and computational infrastructure, nanopore sequencing is becoming an increasingly viable option for clinical diagnostics. The potential to replace multiple conventional assays (e.g., WES, CMA, methylation arrays) with a single comprehensive workflow is particularly appealing for rare disease evaluation (Zhang et al., 2024; Steinicke et al., 2025). In addition, while whole-genome long-read sequencing generates substantial data, ongoing improvements are likely to further streamline data generation and analysis, supporting broader clinical implementation in the near future.

## Conclusion

Our study confirms that nanopore sequencing enables reliable detection of the Hao-Fountain syndrome methylation episignature, alongside pathogenic *USP7* variants, within a single assay. This integrated genomic–epigenomic approach offers a powerful and efficient alternative to traditional multi-platform testing strategies. As nanopore technologies and analytical tools continue to evolve, they hold significant promise for expanding access to precision diagnostics in neurodevelopmental and other rare genetic disorders.

## Data Availability

The datasets presented in this article, including full Oxford Nanopore sequencing profiles and Illumina MethylationEPIC array data, are not readily available because they contain sensitive personal genetic information and cannot be made publicly available under Dutch and EU privacy regulations (AVG/GDPR). In line with FAIR principles, de-identified β-value matrices for the HAFOUS/USP7 episignature are provided in Supplementary Table S1, and additional data can be made available upon reasonable request and following approval of a Data Transfer Agreement and institutional data-protection procedures. Data processing workflows and analysis scripts are available at the Human Genetics Amsterdam UMC GitLab repository (https://gitlab.com/hg-aumc/bioinf/pipelines/sushi). Other requests to access the datasets should be directed to the corresponding author.
