# Supplementary material for: Nanopore sequencing enables combined detection of USP7 variants and a known Hao-Fountain syndrome episignature
Source: Front Genet. 2026 Jan 5;16:1730165. doi: 10.3389/fgene.2025.1730165 (PMC12812390; doi:10.3389/fgene.2025.1730165)
Supplement: Supplementary file 1 [file DataSheet1.pdf]

A

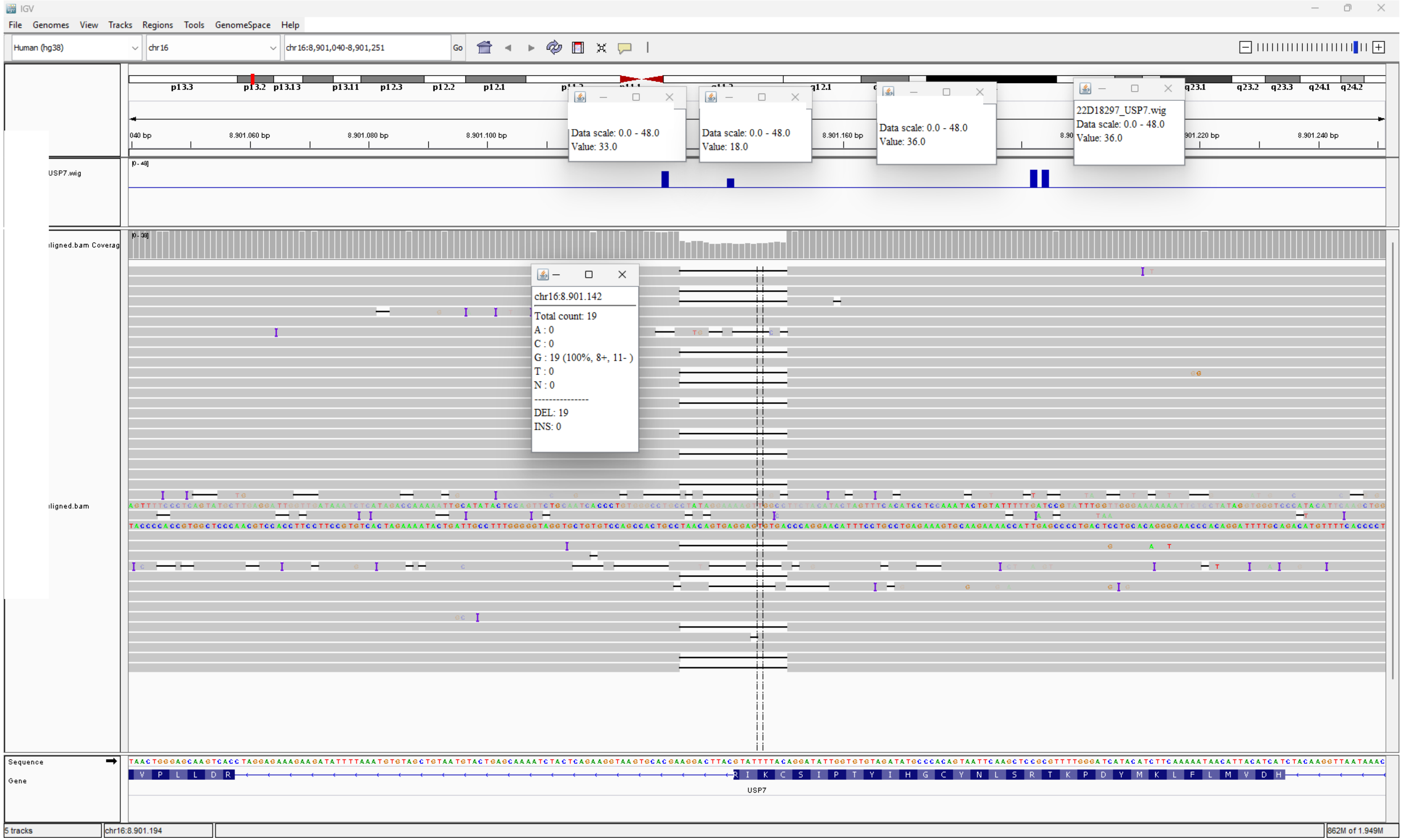

Supplementary Figure 1. IGV visualization of *USP7* variants detected after nanopore sequencing. Shown are representative IGV screenshots from five individuals carrying pathogenic or likely pathogenic *USP7* variants. Variants are highlighted in the sequencing reads as visualized in IGV.

A. Case 1; c.2132_2140+9del

Case 2; c.1988A>C, p.(Glu663Ala) / c.2051A>T, p.(Asp684Val)

B

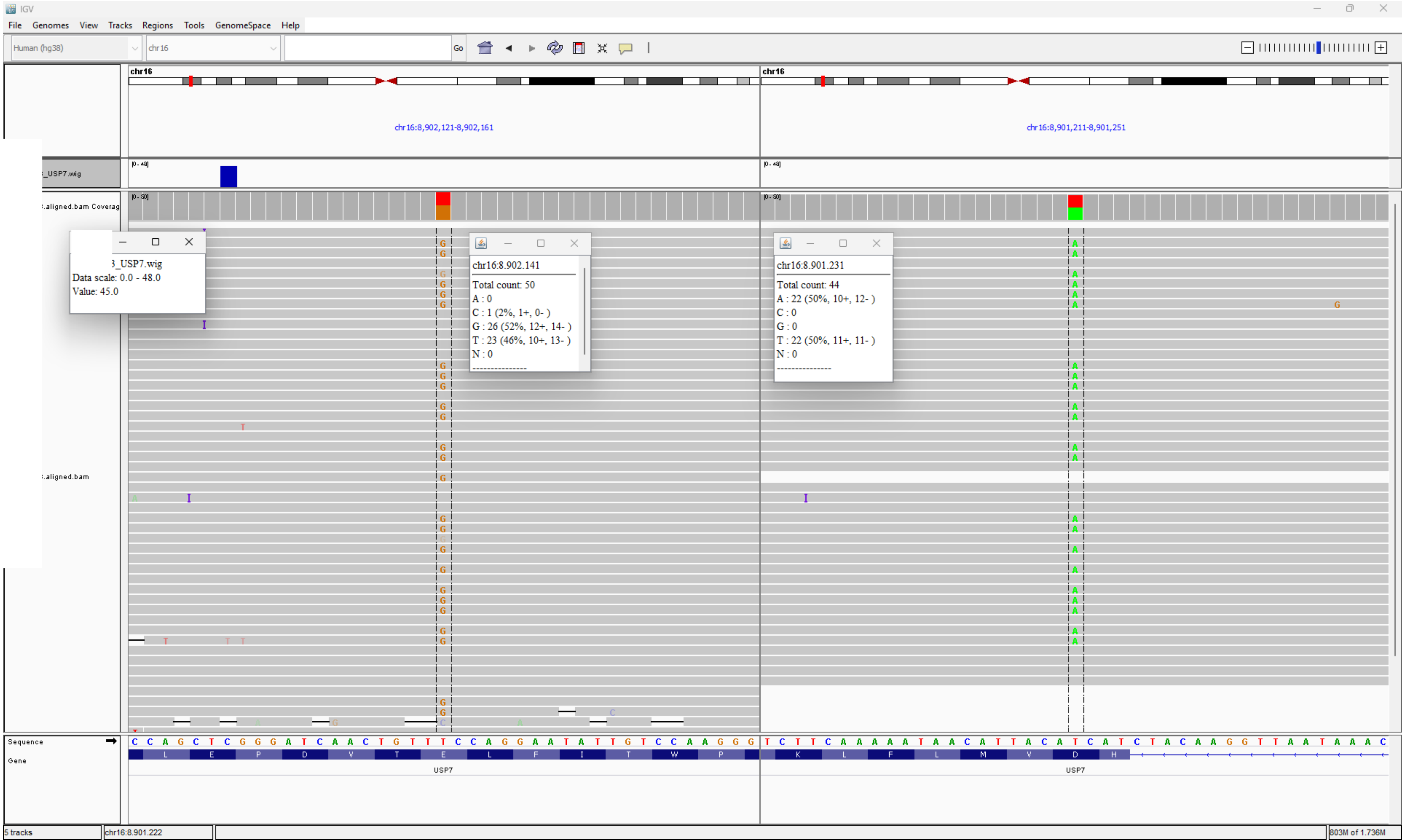

Case 3; arr[GRCh37] 16p13.2(9036896_9283604)x1

C

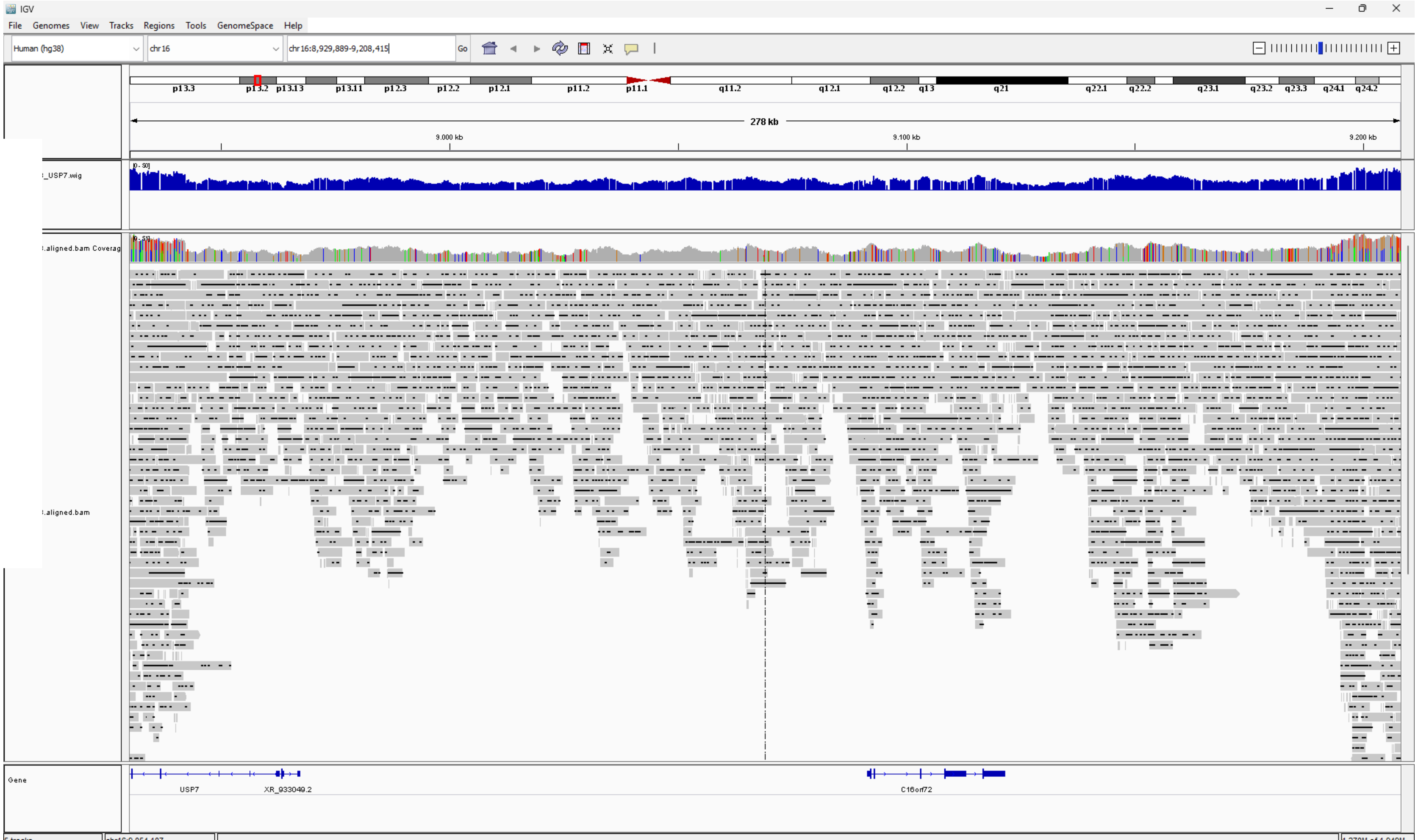

Case 4: c.1258A>G p.(Lys420Glu)

D

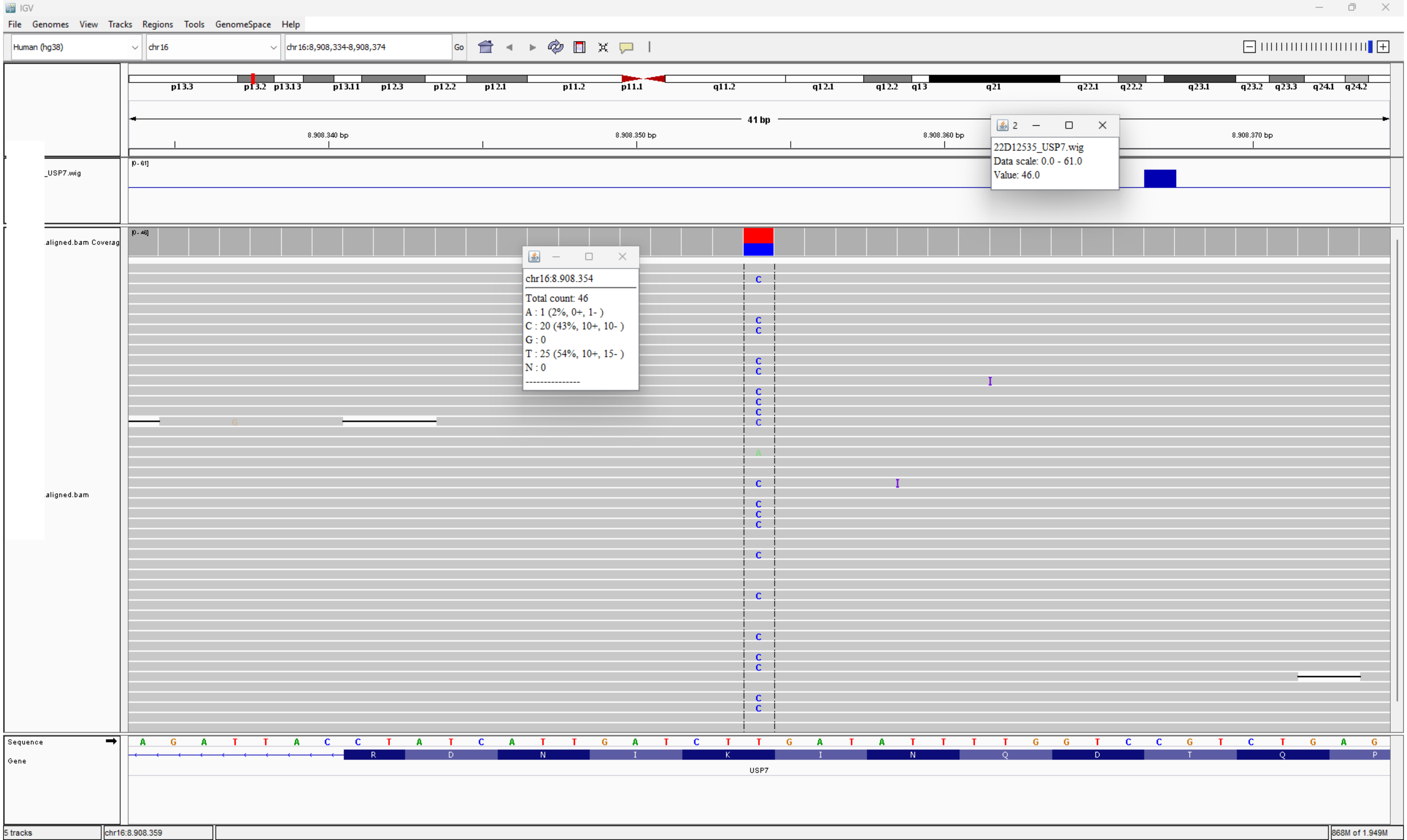

Case 5; c.2232_2235delGAGA p.(Arg745AsnfsTerS)

E

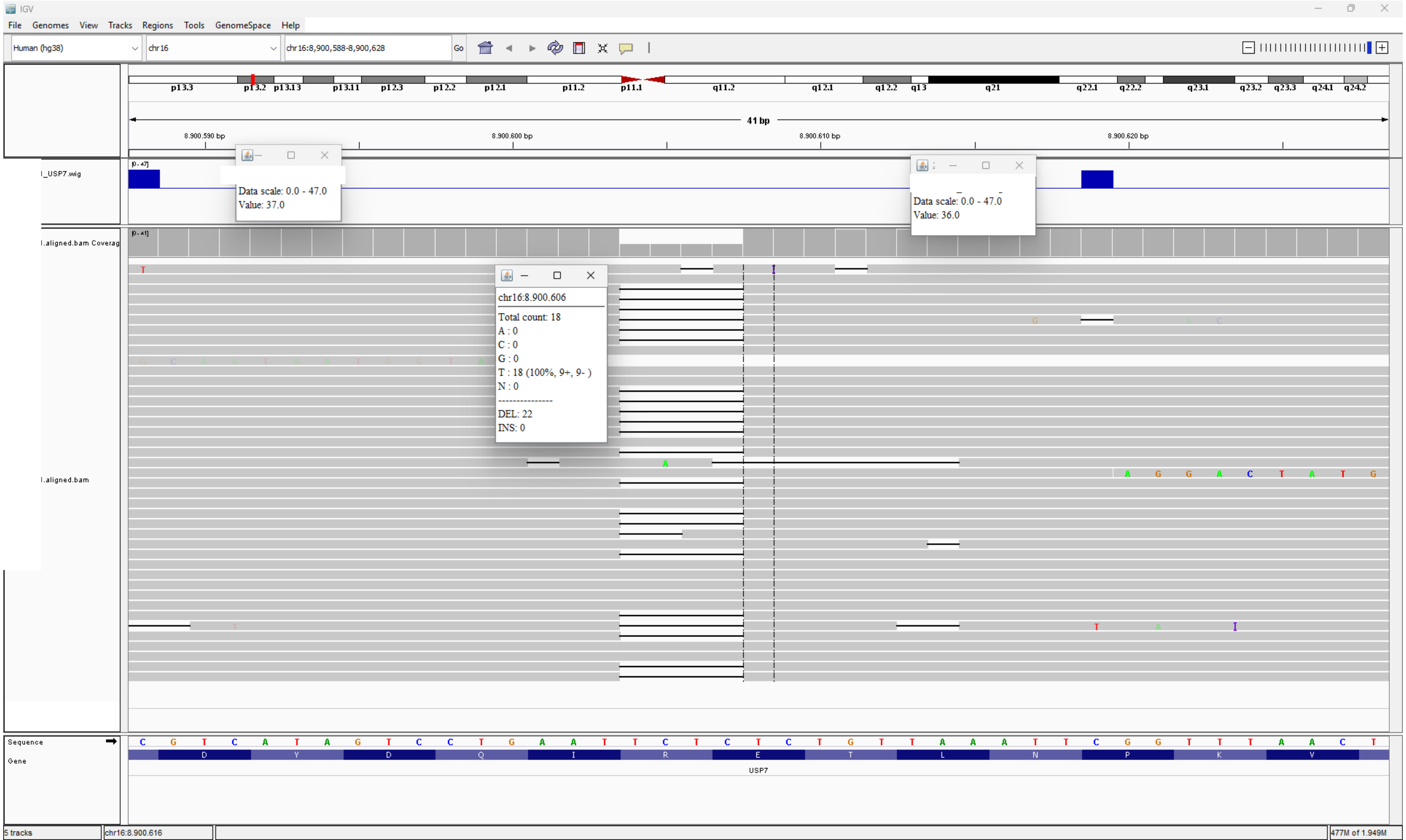

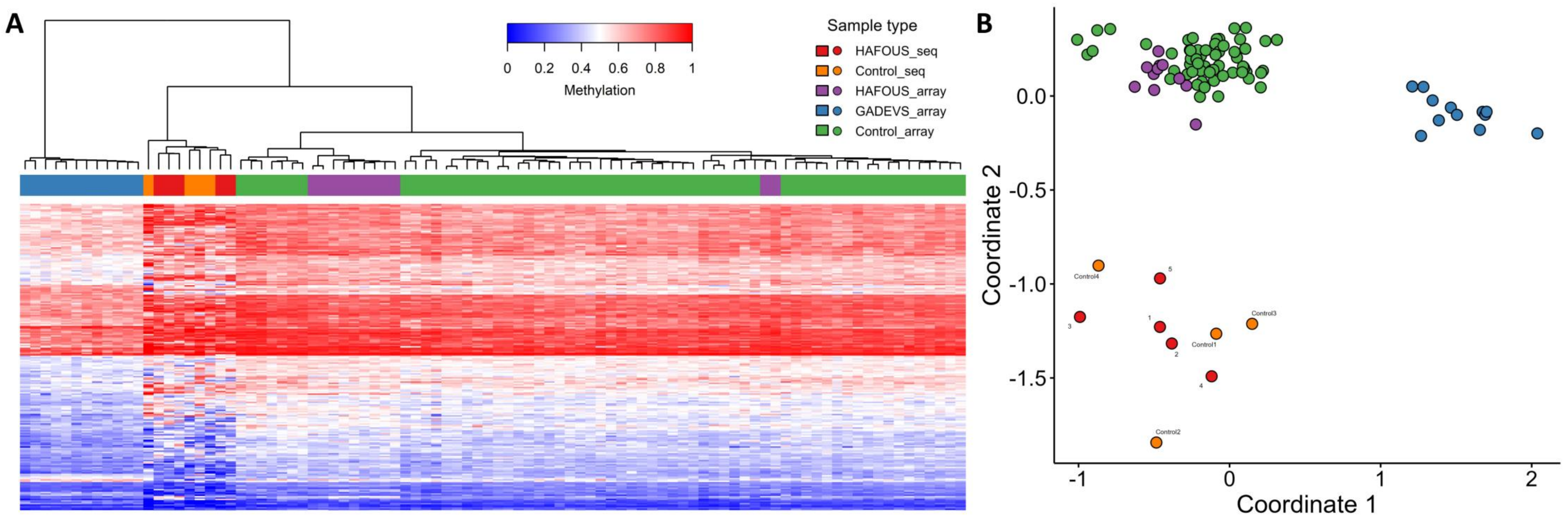

Supplementary Figure 2. GADEVS episignature analysis of sequencing data from peripheral blood in individuals with pathogenic *USP7* variants. A. Hierarchical clustering and B. multidimensional scaling plots show that HAFOUS sequencing cases (red) cluster with Control sequencing cases (orange) when analyzed using a non-HAFOUS (GADEVS) episignature. This pattern mirrors the array data, where HAFOUS array cases (purple) are indistinguishable from Control array cases (green), but distinct from GADEVS cases (blue).
